# ‘Invisible’ orthodontics by polymeric ‘clear’ aligners molded on 3D-printed personalized dental models

**DOI:** 10.1093/rb/rbac007

**Published:** 2022-02-04

**Authors:** Xiaoye Yu, Guanghui Li, Yikan Zheng, Jingming Gao, Ye Fu, Qunsong Wang, Lei Huang, Xiaogang Pan, Jiandong Ding

**Affiliations:** 1 State Key Laboratory of Molecular Engineering of Polymers, Department of Macromolecular Science, Fudan University, Shanghai 200438, China; 2 Department of Orthodontics, Shanghai Ninth People’s Hospital, College of Stomatology, Shanghai Jiao Tong University School of Medicine, National Clinical Research Center for Oral Diseases, Shanghai Key Laboratory of Stomatology & Shanghai Research Institute of Stomatology, Shanghai 200011, China; 3 R&D Center, EA Medical Device Technologies Co., Ltd., Wuxi 214174, China

**Keywords:** 3D printing, polymer, biomaterial, clear aligner, orthodontics

## Abstract

The malalignment of teeth is treated classically by metal braces with alloy wires, which has an unfavorable influence on the patients appearance during the treatment. With the development of digitization, computer simulation and three-dimensional (3D) printing technology, herein, a modern treatment was tried using clear polymeric aligners, which were fabricated by molding polyurethane films via thermoforming on the 3D-printed personalized dental models. The key parameters of photocurable 3D printing of dental models and the mechanical properties of the clear aligner film material were examined. The precision of a 3D-printed dental model mainly relied on characteristics of photocurable resin, the resolution of light source and the exposure condition, which determined the eventual shape of the molded clear aligner and thus the orthodontic treatment efficacy. The biocompatibility of the polyurethane film material was confirmed through cytotoxicity and hemolysis tests *in vitro*. Following a series of 3D-printed personalized dental models and finite element analysis to predict and plan the fabrication and orthodontic processes, corresponding clear aligners were fabricated and applied in animal experiments, which proved the efficacy and biocompatibility *in vivo*. Clinical treatments of 120 orthodontic cases were finally carried out with success, which highlights the advantage of the clear aligners as an esthetic, compatible and efficient appliance.

**Figure:**
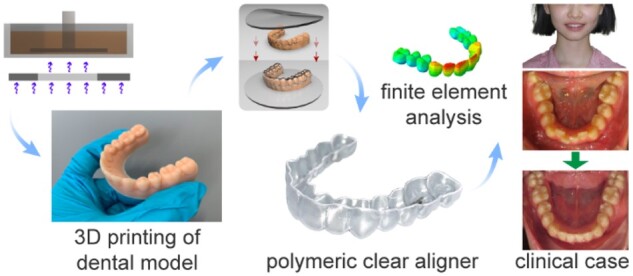

## Introduction

The 3D printing is a flexible additive manufacture method to build personalized 3D structures [1–5]. The methods of 3D printing include fuze deposition modeling, extrusion 3D printing, selective laser sintering and photocuring 3D printing based on digital light processing, liquid crystal display (LCD), stereolithography (SLA) or computer axial lithography. One of the main applications of 3D printing is for tissue engineering and healthcare industry [6–11]. In particular, an important application is in orthodontics, where personalized treatments are much required. As strengthened in this work, 3D printing exerts its advantage well to this personalized medical field.

Wearing clear aligners is a new orthodontic treatment method. Unlike metal braces, clear aligners are made of colorless and transparent thermoplastic polymers, as demonstrated by two clinical cases in a comparative way as in Supplementary Fig. S1. Polymers have been widely used in biomaterials [12–20]. Polymeric clear aligners are particularly helpful for people who pursue esthetics during the orthodontic treatment. Not only can clear aligners help people maintain a good appearance, but also accomplish orthodontic treatment while be propitious to dental health since patients can easily take them off to keep oral hygiene [21–23].

**Figure 1.**
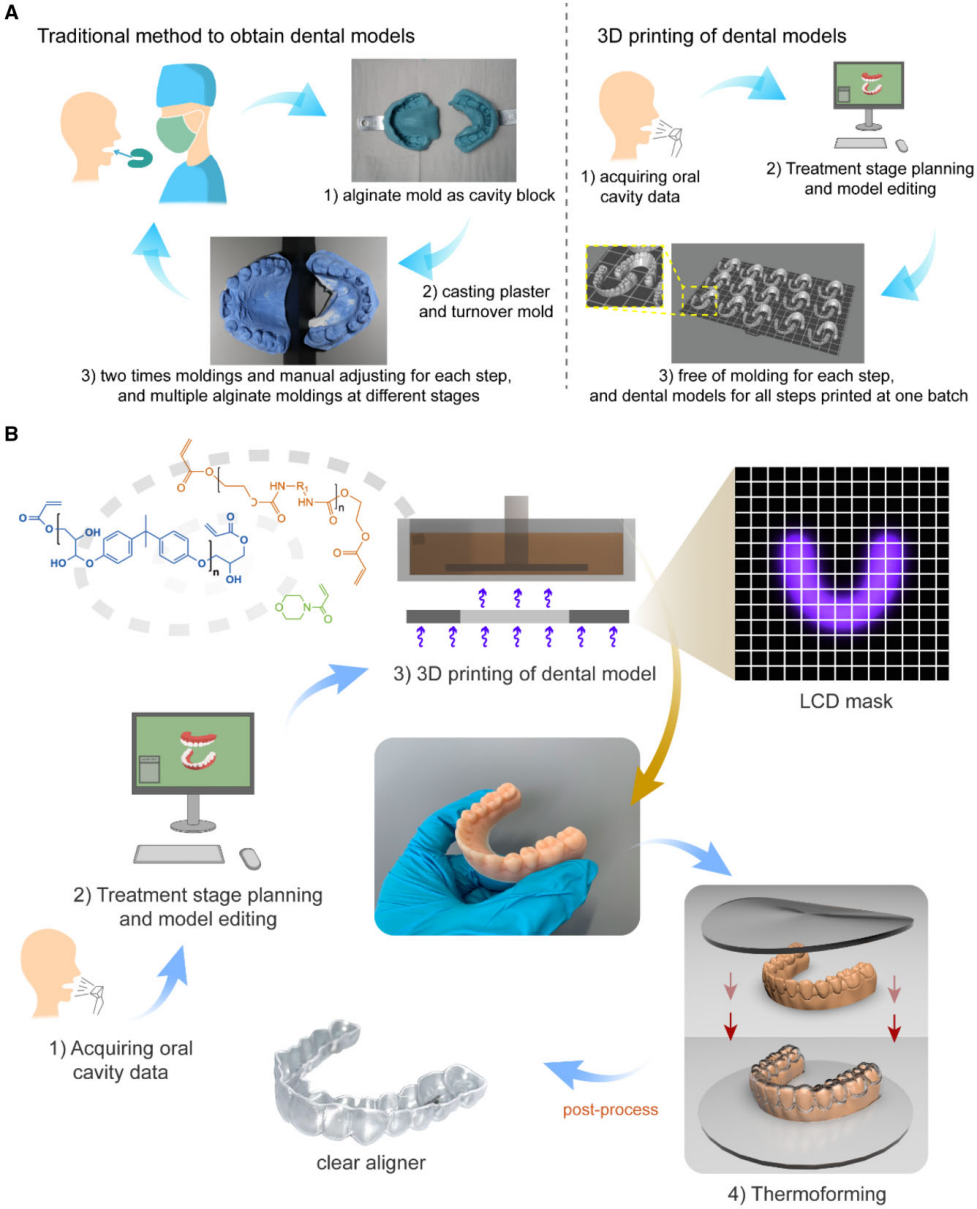
The basic principle of invisible orthodontic aligners. (**A**) Comparison between the traditional plaster casting method and the 3D printing technique to obtain dental models. While plaster casting requires splitting teeth and manual adjusting for each step and multiple cast models at different stages, the 3D printing technique affords mass model manufacture for each step, and dental models for all steps are printed individually yet at one batch. (**B**) The overall procedures to produce clear aligners using 3D-printed models, mainly involving data acquiring, treatment planning, 3D printing of personalized dental models, thermoforming and post processing.

A clear aligner is thermoformed on a teeth mold, and thus it is critical to precisely obtain the mold. As the method to fabricate a dental mold is concerned, one can now select between the traditional plaster casting and the modern 3D printing. In both ways, a doctor needs to plan and adjust the specified teeth of dental models for small incremental movements of each orthodontic treatment step and obtain the solid models. As shown in Fig. 1A, plaster casting in an alginate mold is, as a traditional way, labor intensive and inaccurate; dentists have to split the teeth as isolated tooth unit from the cast model and adjust their position bit by bit manually, also multiple processes of cast models are necessary at different treatment stages. With the development of the 3D printing technology and computer simulation, clear aligner fabrication in orthodontics is embracing a whole new era [24].

Fabrication of clear aligners includes, as shown in Fig. 1B, the following main steps: acquiring the oral cavity data of a patient, orthodontic treatment stage planning and model editing, 3D printing of dental models, thermoforming of a foil or film material to prepare the clear aligner and finally some post-processing steps including trimming along the cutting-line of the clear aligners and labeling them with QR codes by laser. Clear orthodontics requires high precision of clear aligners at each stage to achieve a successful treatment result, and thus the 3D printing process is expected to produce dental models as close as possible to the designed digital models. Not only the characteristics of the photocurable resin should be taken into consideration, but also the parameters set in the printing process are crucial to whether an ideal dental model can be obtained.

In this study, we examined some key parameters of the 3D printing of dental models via a combination of SLA and LCD, and successfully printed the dental models in expected shape. The clear aligners were then molded on the dental models, and the aligner material possessed good mechanical properties. Animal experiments indicated the efficacy of our clear aligners. Finally, a 12-year-old patient case is reported to validate the clinical effectiveness of the polymeric clear aligners.

## Materials and methods

### Acquiring of oral cavity data, planning of treatment stages and model editing

The oral cavity data of a patient was acquired by oral scanning devices (3Shape, Denmark). The scanning is demonstrated in Supplementary Fig. S2. Once the scanning was completed, the data would be further processed by Atreat Processer V3.3.1 before treatment stage planning and model editing. Then, according to each patient’s own situation, we designed incremental movement of target teeth and formed a total orthodontic plan.

### 3D printing of dental models composed of a photocrosslinked resin

The photocurable resin used for 3D-printed dental models was purchased from Shenzhen Anycubic Technology Co., Ltd (China), along with the LCD-SLA 3D printer. One type of resin is indicated in Supplementary Table S1. A cube with side length of 10 mm (*L*0) was designed in Cinema 4D, the STL profiles were loaded into Photon Workshop, where parameters for printing were set. We examined different exposure times (0.5, 1, 1.5, 8 and 20 s) to prepare a modeled cube. Other parameters like layer thickness (0.05 mm) were kept the same for the series of cubes. After printing, models were washed with alcohol. The sizes of cubes (*L*) with different exposure times (*n *=* *3 for each group) were measured by a digital caliper with an accuracy of 0.01 mm. Cubes with the side lengths of 15 and 20 mm were designed and processed in the same way, the exposure time was set at 8 s to investigate the relationship between the exceeded length and the theoretical size of the models. After an investigation of proper exposure time, the digital dental models from Angelalign company were 3D-printed.

### Characterization of the film material for clear aligners

For convenience, the film material in our study, thermoplastic polyurethane based on 4,4'-diphenylmethane diisocyanate and 1,6-hexanediol, is abbreviated into thermoplastic polyurethane (TPU). ^1^H and ^13^C nuclear magnetic resonance (NMR) were recorded with a Bruker type AVANCE III HD 400 MHz spectrometer, where chemical shifts for ^1^H NMR spectroscopy were determined with respect to non-deuterated solvent residues, DMSO-D6 (δ 2.50), and those for ^13^C NMR spectroscopy were determined with respect to DMSO-D6 (δ 39.52). Differential scanning calorimeter (DSC, TA Q2000, USA) was used to analyze the thermal properties of the TPU. The sample was of weight about 6 mg. In DSC scanning, TPU was kept at −50°C for 3 min, and then heated to 250°C and maintained for 3 min; after that, the sample was cooled to −50°C and kept for 3 min, and then heated to 250°C again. The heating and cooling rates were both set as 10°C/min. The measurements were carried out under nitrogen atmosphere. The molecular weight of the TPU film was tested with gel permeation chromatography (GPC) (Agilent/Wyatt, USA) using N,N-dimethylformamide as eluent and a series of polystyrene with known molecular weight as standard. Also, the TPU film was detected via Fourier transform infrared spectroscopy (FTIR) (Nicolet 6700, USA) using the mode of attenuated total reflection.

The mechanical properties of the TPU film were tested in a universal testing machine (Instron 5943, USA). The stretching rate was 1.0 mm/min in the tensile tests in water bath at 37°C. In the right-angle tear tests, testing speeds were from 10 to 500 mm/min. The tear strength *T*_s_ is defined by

(1) $$T_{s}=\frac{F}{d}.$$

Here, *F* is the maximum force in units of Newton, *d* is the median thickness of the test sample in units of millimeter. The testing speed was 0.5 mm/min in the 3-points bending test. In the stress–relaxation tests, the samples were immersed in water bath at 37°C for 24 h, then stretched to 2% nominal strain at a rate of 0.5 mm/min and held there constantly in water bath at 37°C.

### Modeling of the thermoforming procedure to fabricate clear aligners

The thermoforming process was simulated using the finite element method (Abaqus, Dassault Systèmes Simulia Corp., USA). In the modeled thermoforming process, the circular splint was simulated using triangular shell elements S3R. The pressure of 400 kPa was introduced on its upper surface at 200°C. The splint was forced by the pressure onto the rigid teeth model to replicate the shape of the teeth model. The redundant elements below the cutting-line, which covered the gingiva, were removed as in practice to obtain the aligner. After that, the aligner model could be used in other simulations, such as the calculation of the orthodontic forces and moments, and numerical experiments of the aligner detaching from the jaw.

### Cell experiments

Cytotoxicity tests were carried out in the extracted fluid of the TPU film with a cell counting kit-8 (CCK-8, Dojondo, Japan) assay. Mouse embryonic fibroblasts (NIH/3T3, purchased from the Cell Bank of the Chinese Academy of Science) and human epidermal keratinocytes (HaCaT, Shanghai Kang Lang Biological Technology Co., Ltd) were used as the two model cell types.

Dulbecco’s modified Eagle medium (Gibco, USA) containing 10% fetal bovine serum (Gibco, USA) and an additional 100 U ml^−1^ of penicillin (Gibco) and 100 μg ml^−1^ of streptomycin (Gibco) was used to extract the TPU film with a 3 cm^2^/ml ratio (prior to thermal forming) at 37°C for 24 h according to ISO 10993-12: 2012.

The cells were seeded in 96-well plates at a density of 5000 cells per well with 100 μl of culture medium and incubated at 37°C in 5% CO_2_ atmosphere for 24 h before use. The group of cells with culture medium with 0.1 mg/ml sodium dodecyl sulfate (SDS) was set as the positive control of cytotoxicity or the negative control of cell viability (0%); the group of cells with culture medium was set as the negative control of cytotoxicity or the positive control of cell viability (100%). Then, the culture medium was replaced by the extracted fluid, SDS or culture medium. After culturing for 1–3 days, CCK-8 tests were performed by applying a new culture medium with the ratio of complete culture medium and CCK-8 9:1, then the absorbance of each sample was measured at 450 nm.

We also used 4′,6-diamidino-2-phenylindole, dihydrochloride (Invitrogen, Thermo Fisher Scientific, USA) and rhodamine phalloidin (Sigma) to stain the nucleus and F-actin of cells, respectively. After a 3-day culture, the cells were stained and observed in a fluorescence microscope (Zeiss, Germany).

### Hemolysis tests of the TPU film

A hemolysis experiment was conducted to judge the hemolysis extent of the TPU film for clear aligners. In the test group, 2.5 g TPU film was put into 5 ml physiological saline per tube. In the negative group, only 5 ml physiological saline was used per tube. In the positive group, 5 ml pure water was used per tube. We prepared three tubes for each group. All the tubes were kept in a 37°C environment for 30 min. After that, 2 ml fresh anticoagulant-treated rabbit blood was diluted by 2.5 ml physiological saline, and 0.2 ml diluted rabbit blood was put into each tube. Then, all the tubes were kept in a 37°C environment for another 60 min followed by pouring out the fluid into centrifuge tubes. The centrifugation at 800 *g* lasted for 5 min. The absorbance of the supernatant was tested in a spectrophotometer at a wavelength of 545 nm. The hemolysis rate of the TPU film was calculated by

(2) $$\mathrm{Hemolysis}\;\mathrm{rate}=\frac{A-B}{C-B}\times 100\%.$$

Here, *A*, *B* and *C* are the absorbances of the test, negative and positive groups, respectively.

### Teeth movement in a rabbit model

As an experimental animal, the rabbits have dentition that could meet the requirements of no gap between the upper central incisors, no early contact of anterior teeth, no tooth disease and no loose teeth. The animal experiments were approved by Ethics Committee, Shanghai Ninth Peoples’ Hospital.

We examined three rabbits. Their lower incisors were moved distally and horizontally and separated to produce a gap by wearing clear aligners specially designed and fabricated personalized to each rabbit. The experiment was designed to move the teeth 0.2 mm per 4 days. Every 4 days, before a new clear aligner was applied on each rabbit, we obtained a mold of the alginate calcium hydrogel using the rabbit teeth as template, and then carried out the plaster casting using the alginate mold as template, and finally measured the distance between the lower incisors in the plaster mold.

### Clinical cases

This article reports 120 clinical cases. Besides the statistics of these cases, an orthodontic case of a 12-year-old female patient treated with the clear aligners is demonstrated in details. The clinical treatments were approved by Ethics Committee, Shanghai Ninth Peoples’ Hospital. Informed consent was obtained from the patient and her parents, and principles outlined in the Declaration of Helsinki were followed.

She is an Angle Class I crowding case. The girl was treated by expanding the upper and lower arch with a series of clear aligners to align her teeth. The arch expansion effects were mostly achieved by buccolingual inclination. The orthodontic treatment by wearing clear aligners was designed in 38 steps (2 weeks per step). All of the 38 pairs of clear aligners were molded by corresponding 3D-printed personalized dental models.

### Statistical analysis

All the data were treated by ANOVA analysis and are shown as mean ± standard deviation. It is considered to have a significant difference when the *P*-values is <0.05. The differences are marked as ‘*’ for 0.01 < *P *<* *0.05, ‘**’ for 0.001 < *P *<* *0.01 and ‘***’ for *P *<* *0.001.

## Results

### Acquiring oral cavity data and designing treatment stages and models

The oral cavity data were processed mainly to separate every single tooth from each other (Supplementary Fig. S2), so that further movement design could be performed. One of the cases of the total treatment stage planning and model editing is shown in Supplementary Fig. S3. Only a small incremental movement was set for each treatment step.

### 3D printing of dental models with optimized exposure time

**Figure 2.**
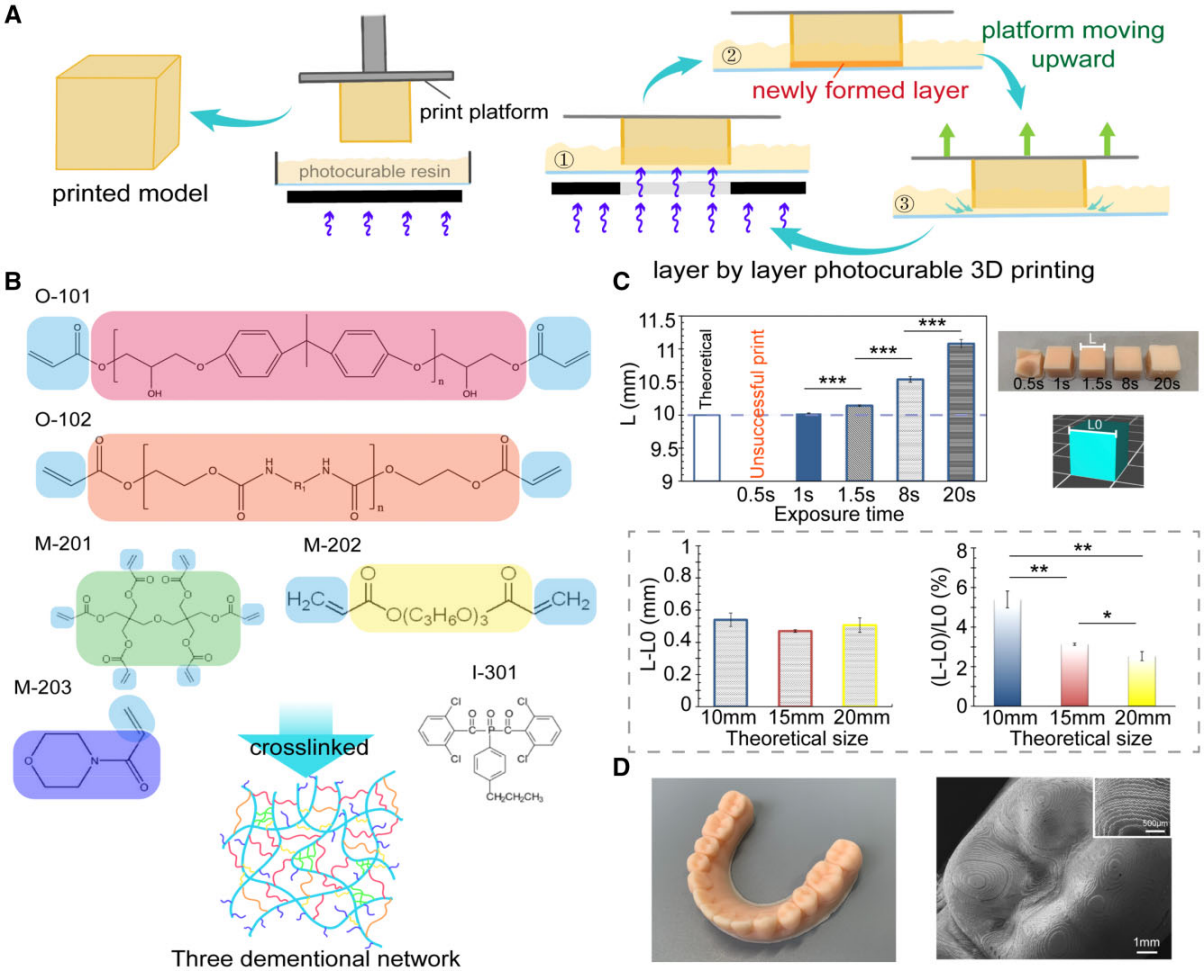
3D printing via SLA to generate molds to eventually fabricate polymeric invisible orthodontic aligners. (**A**) Schematic diagram of the principle of 3D printing process with an LCD-SLA-type 3D printer. (**B**) Chemical structures of the photocurable resin components listed in Supplementary Table S1. (**C**) Histograms about the relation between the length of cubes and exposure time (*n *=* *3). Photographs show the cubes with a theoretical length of 10 mm, which were obtained via printing with different exposure time. Diagraphs in the dotted frame show the absolute and relative deviations of lengths of cubes with different theoretical sizes at an exposure time of 8 s. (**D**) A global view and SEM image of a 3D-printed polymeric dental model.

The accuracy of a 3D-printed dental model has a deterministic effect on the resultant clear aligner. Combination of LCD and SLA can afford an appropriate 3D printing method to generate a dental model. The general principle of an LCD-SLA-type 3D printer is based on layer-by-layer exposure in a preset time to solidify certain images of each layer, as shown in Fig. 2A. The chemical structures of the photocurable resin components are presented in Fig. 2B. Before the 3D printing of dental models, parameters should be set for using a photocurable resin in a 3D printer. In Fig. 2C, the cube with a theoretical size (side length) of 10 mm was unable to form completely with an exposure time of 0.5 s. When the exposure time was set at 8 s, although the cube could form completely, the side length (*L*) of the cube exceeded about 5.5% over the theoretical one (*L*0). When exposure time reached 20 s, the cube started to form an irregular shape because the LCD mask could not sufficiently block the light in the unwanted area due to its own construction, and the resin was crosslinked to some extent in the unwanted area and may form some thin films to interfere with the expected regular printing process.

We also examined the extent of size exceeding in models of different sizes. Cubes with theoretical sizes of 10, 15 or 20 mm were 3D-printed with an exposure time of 8 s. The results showed that the absolute difference *L*−*L*0 did not change significantly among models with different sizes, so the relative value (*L* −*L*0)/*L*0 decreased along with the increase of model size, which means that the size exceeding effect is mild in 3D printing a large model.

The cube with an exposure time of 1 s had the closest size to 10 mm (Fig. 2C). However, the printing failed sometimes under such a short exposure, especially when the digital model was set at a low filling rate inside. At last, we found out that the exposure time of 1.5 s was most suitable in our system. Based on our capturing of the main controlling conditions of such a 3D printing method, we successfully 3D-printed a dental model, as demonstrated in Fig. 2D. The resin model obtained via 3D printing served as the mold to fabricate the eventual clear aligner via thermoforming of a TPU foil or film.

### Characterization of the film material for clear aligners

**Figure 3.**
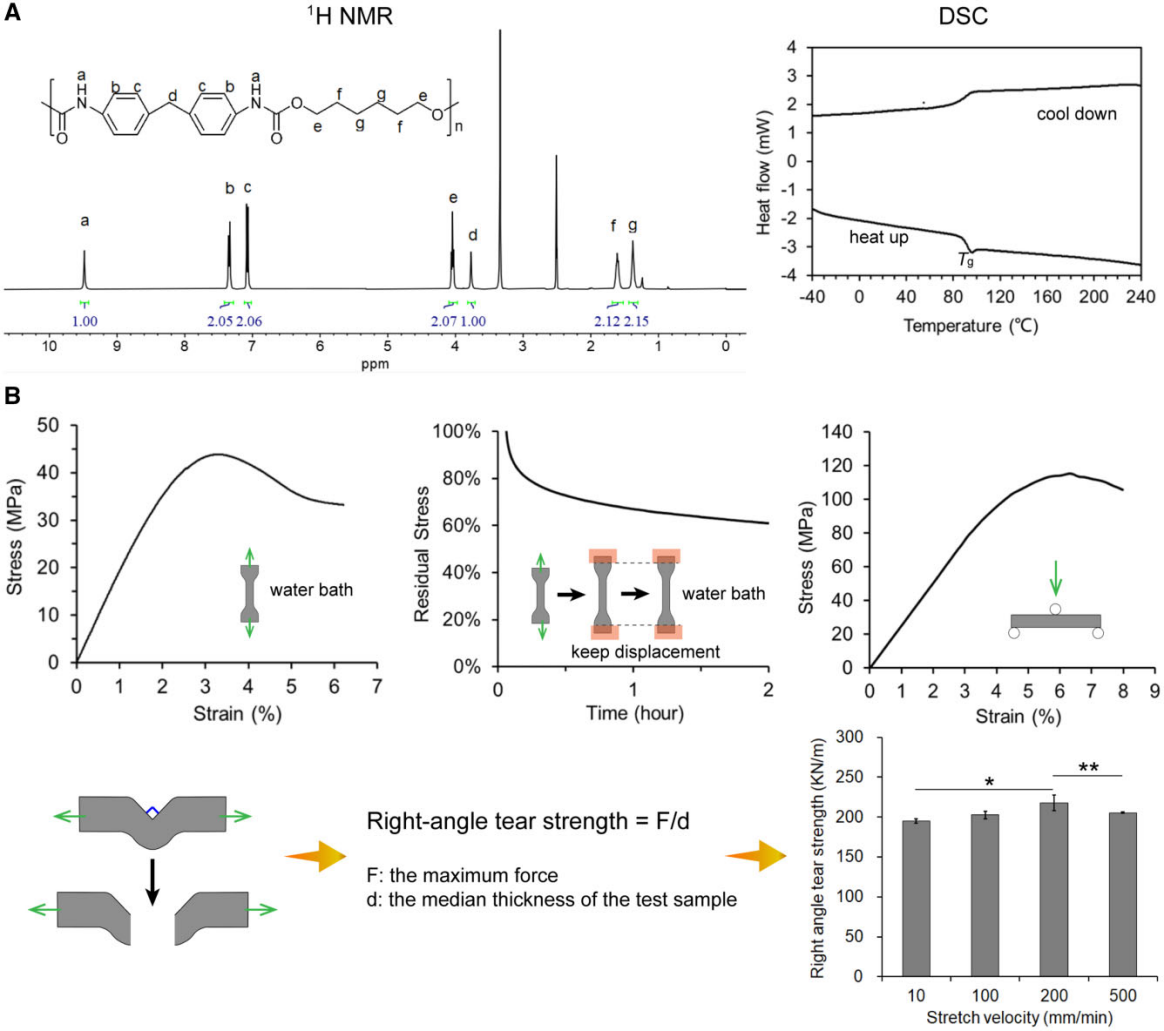
Characterization of TPU as the raw material of the clear aligners. (**A**) ^1^H NMR spectrum of TPU in DMSO-d_6_ and DSC curves (the cooling down curve and the second heating up curve) of the clear aligner film material. (**B**) The mechanical tests of the clear aligner film material: tensile test, stress-relaxation test, three-point bending test and right-angle tearing test.

To illustrate the applicability and characteristics of the TPU film, we conducted a series of tests. The NMR results shown in Fig. 3A (^1^H NMR) and Supplementary Fig. S4 (^13^C NMR) indicated the chemical structure of the TPU, and the DSC curves indicated that the glass transition temperature was about 90°C. As shown in Supplementary Fig. S4, the weight-average molecular weight of the polymer was, according to the GPC experiment, about 3.17 × 10^5^, and molar mass dispersity, namely, weight-average molecular weight over number-average molecular weight was about 2.3. We performed FTIR measurements, and the spectrum confirmed the characteristic peaks of the TPU (Supplementary Fig. S4).

We further examined the mechanical properties of the polymer film. The results of tensile tests of the TPU film are shown in Fig. 3B. The yielding stress was 43.8 MPa and yielding strain was 3.4%. The tensile modulus was about 1950 MPa. As a typical viscoelastic property of a polymeric material, the stress–relaxation behavior of the TPU film was also examined in water bath at 37°C, and the residual stress at 2% strain remained above 60% in 2 h. The three-point bending test determined a high bending modulus of 2511 MPa of the TPU film. The right-angle tear strength of TPU film was around 200 KN/m at examined stretch velocities (Fig. 3B).

### Thermoforming procedure to fabricate clear aligners

**Figure 4.**
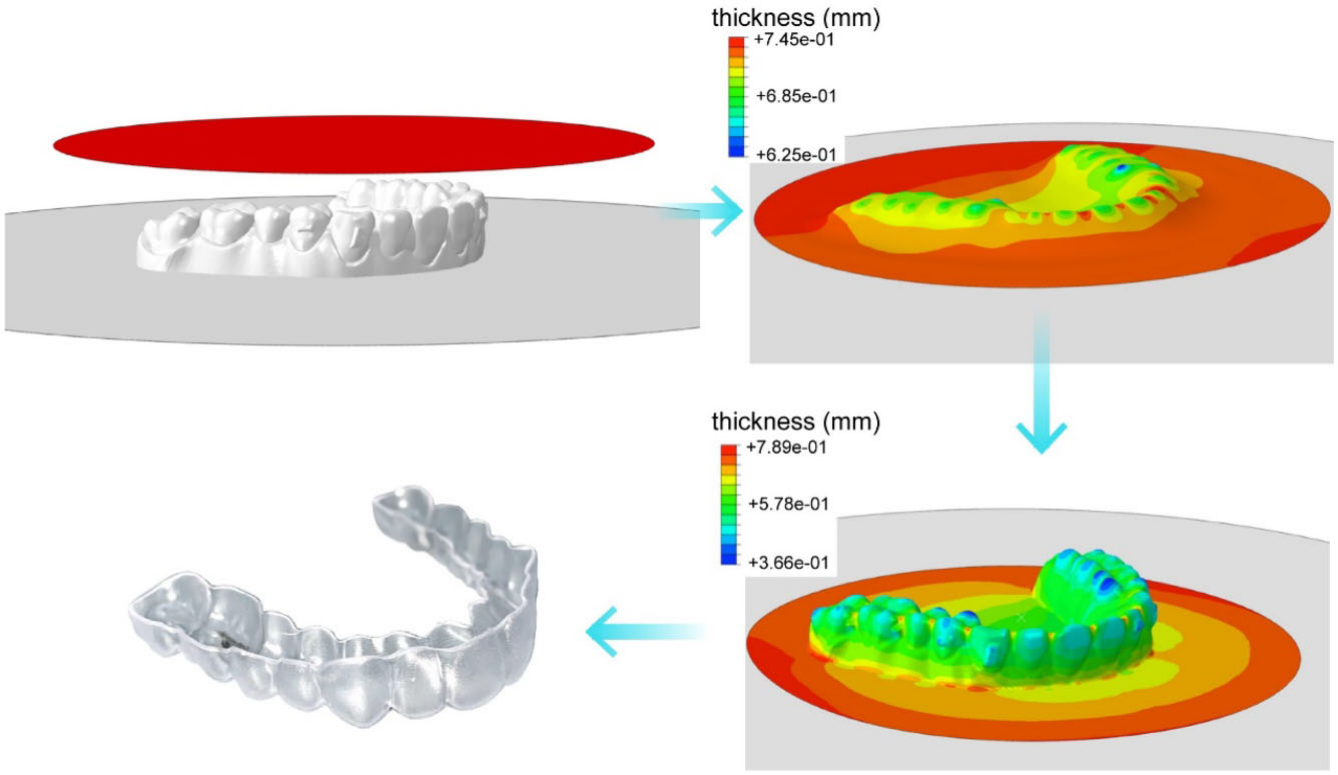
Finite element analysis of the thickness of the film material during the thermoforming process to form a clear aligner (upper left and upper/lower right) and an experimental result (lower left).

The clear aligners were formed by molding on the dental models that were 3D-printed before. The original thickness of the TPU film was about 0.75 mm. When heated up and compressed onto the 3D printed dental model, the film on the teeth region started to get thinner. The simulation outputs from finite element analysis and an experimental result are shown in Fig. 4. At the final stage, the overall thickness of the clear aligner was approximately uniform. The resultant aligners were, except some thinner parts at the cusp of teeth, mechanically reliable for orthodontics.

### Biocompatibility of the TPU film

**Figure 5.**
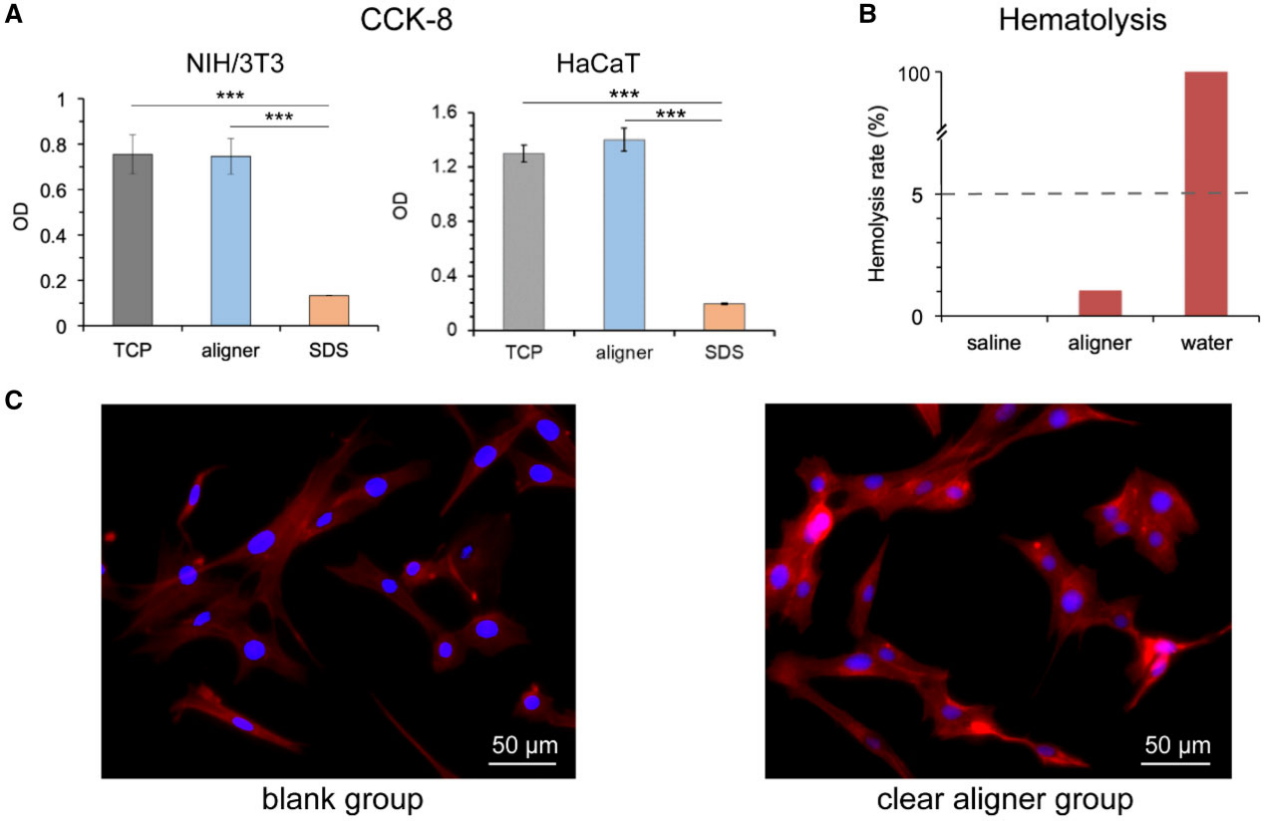
*In vitro* biocompatibility of the aligner materials. (**A**) CCK-8 test of cell viability of the clear aligner film material (TCP: tissue culture plate; aligner: foil or film material to thermoform a clear aligner; SDS: sodium dodecyl sulfate. For each group, *n *=* *4). (**B**) The hematolysis experiment of the film material of the clear aligner. (**C**) Fluorescence photograph of cells cultured in the culture medium (blank group) and the extracted fluid of clear aligner film (clear aligner group).

The biosafety and cell–material interactions are critical for any application of a medical material [25–33]. To examine the safety of our clear aligners, we carried out some *in vitro* biological experiments. As shown in Fig. 5A, CCK-8 assay proved the good biocompatibility of the TPU film, no significant difference was found between the negative group (tissue culture plate) and the experimental group (clear aligner film) in the cytotoxicity test for both types of cells. In the hemolysis experiments (Fig. 5B), the calculated hemolysis rate was about 1.04%, which met the criterion of lower than 5%. The fluorescence photographs of NIH/3T3 cells after a 3-day culture in the culture medium and the extracted fluid of the TPU film both showed normal cell morphology (Fig. 5C).

### Teeth movement experiments in a rabbit model

**Figure 6.**
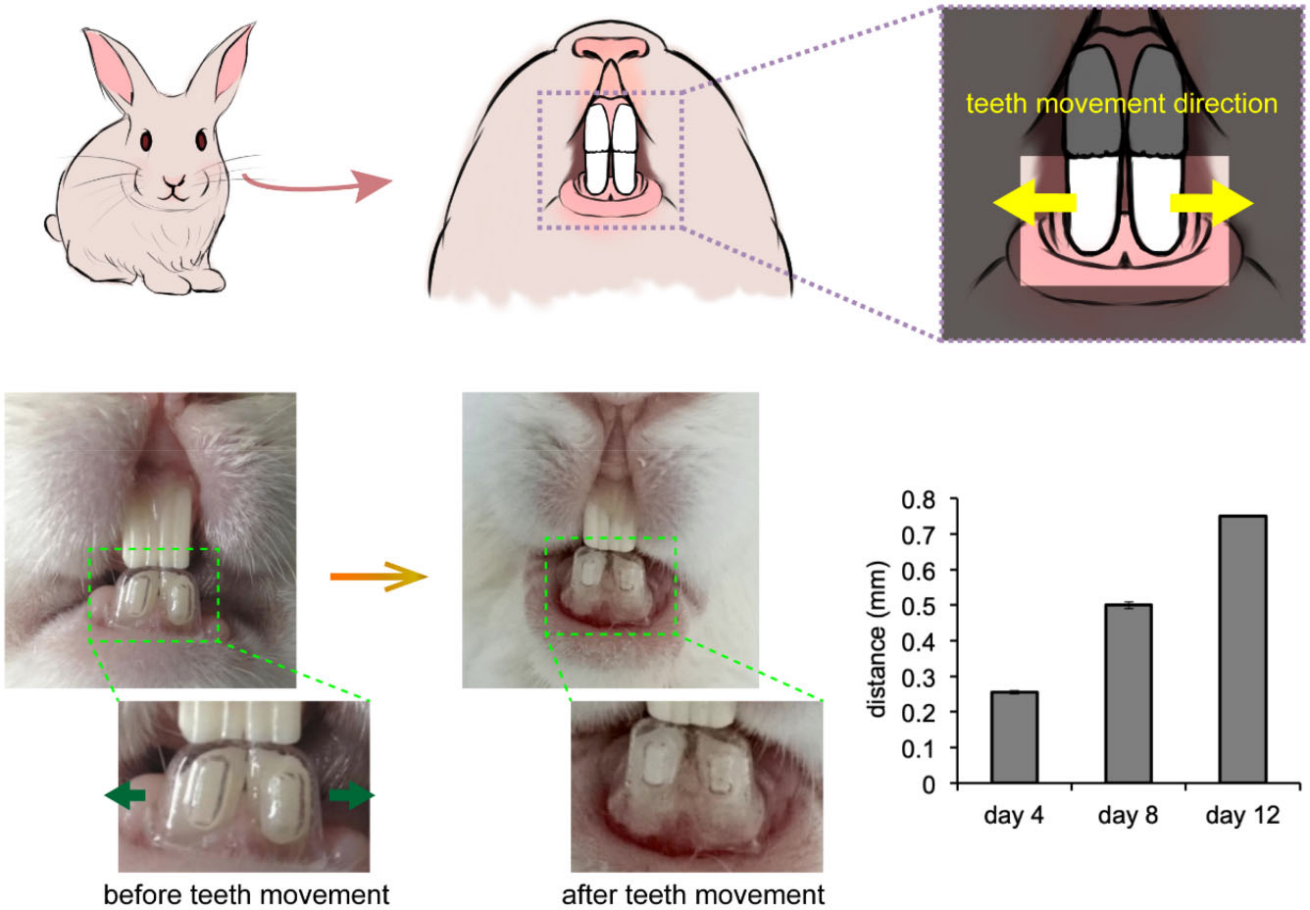
Teeth movement experiment by applying polymeric clear aligners on rabbits. Distance between the lower incisors kept enlarged during the treatment process.

To investigate the capability of the clear aligner to enable teeth movement, we designed an experiment on the teeth of rabbits to make the lower incisors move apart from the middle. As shown in Fig. 6, we had the rabbits wear clear aligners that were individually made for them. The animal experiment was performed to move the teeth 0.2 mm per 4 days. Every 4 days, we measured the distance between the lower incisors, and the distance between the lower incisors was found to grow within time. Since rabbits are rodents, their incisors would keep growing and grinding during their lives, and it is normal that the appearance of the incisors of a rabbit seems not identical after some time. From Fig. 6, the low incisors that were ‘protected’ by the aligners from abrasion looked similar before and after the teeth movement; the upper incisors, however, seemed change in their shape because of the constant grinding of the rabbit. The examined rabbits exhibited an obvious gap between the lower incisors after teeth movement generated by the polymeric aligners.

### Clinical orthodontic treatment

In our study, 120 cases were treated with the clear aligners. These patients were faced with the problems of crowding, space, deep bite, excessive overjet and cross bite, as listed in Table 1. All of the 120 cases were corrected and well aligned.

**Table 1.** The classification of the 120 clear aligner treatment cases

Type	Number	Note	Fraction (%)
Crowding	67	The sum of the crown width larger than the length of the existing arc of the dental arch	55.8
Space	15	Space or gap between teeth	12.5
Deep bite	26	The maxillary anterior teeth cover more than one-third of the labial surface of the mandibular anterior teeth or the cutting edge of the mandibular anterior teeth bites more than one-third of the lingual surface of the maxillary anterior teeth	21.7
Excessive overjet	3	The horizontal distance from the cutting edge of maxillary incisor to the lip surface of mandibular incisor exceeds 2 mm	2.5
Cross bite	9	When occluding, the lingual surface of the mandibular anterior teeth covers the labial surface of the maxillary anterior crown	7.5

**Figure 7.**
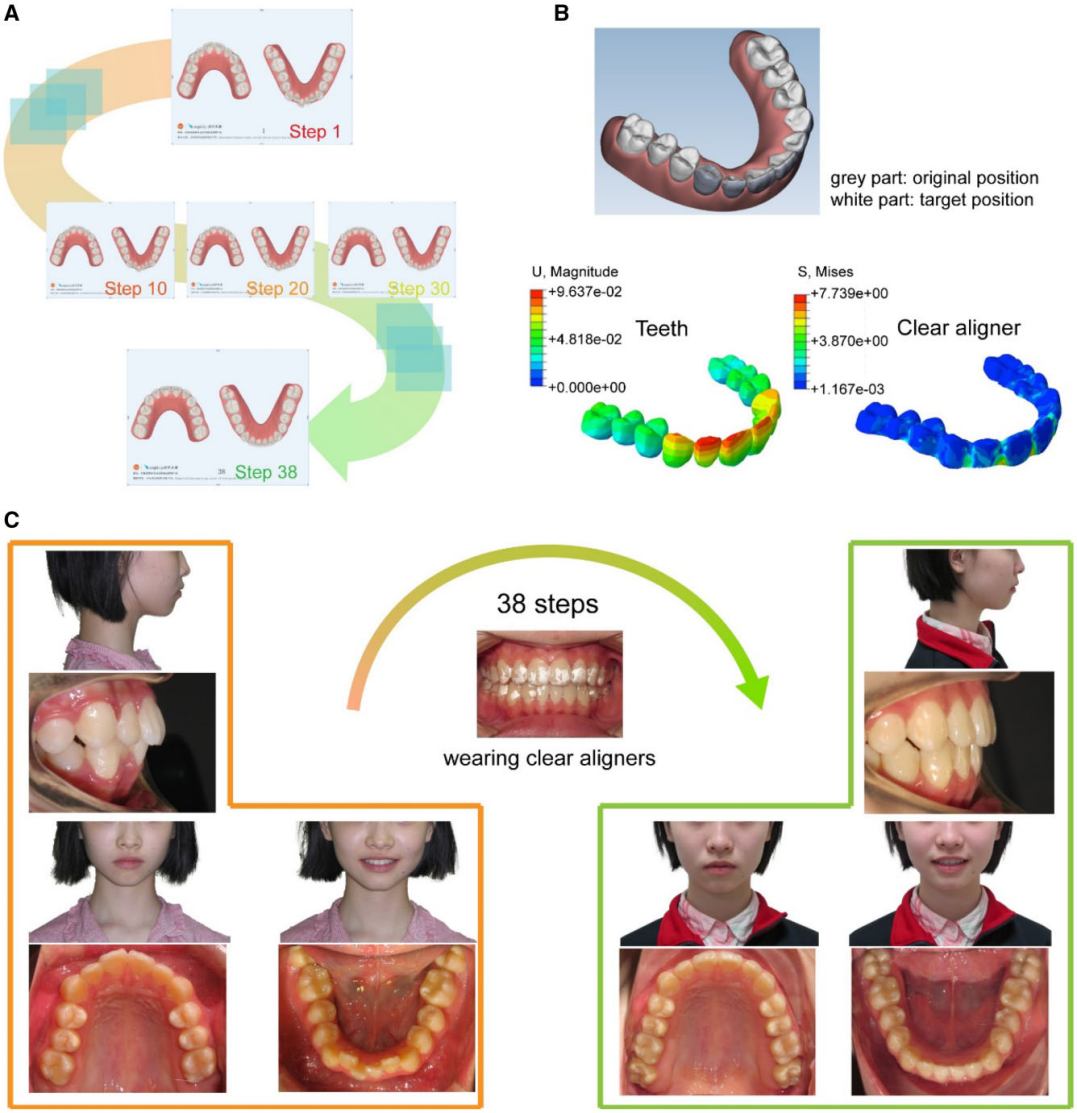
A clinical case of a 12-year-old girl to be treated by the clear aligners. (**A**) 5 out of all 38 steps of computer virtual designs of the orthodontic treatment case of the patient by wearing clear aligners. (**B**) Computer simulation of one step of teeth movement design. The gray part shows the original position of her teeth; the white part shows the target position of her teeth. Finite element analysis of the teeth displacement and the stress distribution in the clear aligner at the corresponding step. (**C**) Photographs to show improvement in alignment of the patient’s teeth after all 38 steps of the orthodontic treatment.

Most of the cases were crowding cases, and here, we report a typical crowding case with results shown in Fig. 7. This is an orthodontic case of a 12-year-old female patient treated with the clear aligners. She used to suffer from an Angle Class I crowding. The girl was treated by expanding the upper and lower arch with a series of clear aligners to align her teeth. Before 3D printing of dental models, a series of orthodontic steps were designed according to the patient’s own condition. As a demonstration, all of the 38 steps were arranged for this case are presented in Supplementary Fig. S3, and 5 out of all 38 orthodontic steps are shown in Fig. 7A. The prediction of teeth movements and stress distribution on the matched clear aligner of a specific step among them by finite element analysis are indicated in Fig. 7B, where her teeth were designed to move from the original position (gray part) to the target position (white part) in the step.

The teeth of the patient improved a lot after the 38-step orthodontic treatment (Fig. 7C), indicating the effectiveness of our orthodontic system. Photographs from other angles of view are presented in Supplementary Fig. S5. The patient wearing clear aligners is shown in Supplementary Fig. S6. All of these results illustrated that the polymeric clear aligners worked well.

## Discussion

Orthodontic treatment enhances the esthetics and functionality of teeth by using appliances that can exert force to enable effective teeth movement, but are, based upon the classic metal braces, usually visible and thus affects beauty [22]. In contrast, invisible orthodontics adopts transparent polymer materials to fabricate clear aligners that can both achieve orthodontic task and reserve the esthetics for people who are going through the orthodontic treatment (Fig. 1). Clear aligners make orthodontic treatment easier to be accepted by reducing the psychological burden of people because of their comfort and elegancy. Compared to traditional brace methods, the clear aligner treatment makes it easier for people to maintain oral hygiene and affects the health of periodontal tissue [23]. With the help of computer simulations, the whole clear orthodontic treatment process can be planned step by step predictably [21, 24]. Tooth alignment treated by buccolingual inclination of upper and lower incisors can produce satisfied clinical results [34]. Previously, a clear aligner was not effective enough for a complex force system to result in an intended tooth movement [35], because it was not as strong as braces in producing adequate occlusal contacts, controlling teeth torque and retention [36]. So it is desired to improve the overall usability of clear aligners, in particular, in the aspects of materials and aligner design.

3D printing nowadays is under rapid development in medical fields. As for clear orthodontics, it enables the personalized fabrication of dental models of each step with high efficiency and precision. Moreover, a series of aligners could be fabricated at one time, which significantly improve the fabrication efficiency. In contrast, the traditional method to obtain dental models and produce clear aligners are tedious and inaccurate, which requires patients to visit the dentists repeatedly during the orthodontic process, while for 3D printing, the visiting time could be largely saved and a more predictable and convenient orthodontic treatment is presented to patients.

The fidelity of 3D printing has always been a fundamental target for researchers to achieve [37, 38]. The free radical polymerization in a liquid material during an over-exposure time may lead to an exceeding of size over the designed one, while an insufficient exposure cannot guarantee shaping. So it is important to find out appropriate 3D printing parameters prior to use. Especially for orthodontic treatment, where precise medicine is much demanded, the fabrication that involves 3D printing should be carefully examined [39–42]. Herein, we captured the key parameters for the 3D printing of a resin mold using the LCD-SLA strategy, and thus successfully generated the polymeric dental mold (Fig. 2).

While the principle of a clear aligner has been justified, better mechanical properties of the foil or film materials are expected in order to maintain the orthodontic force for a long time and achieve the expected position according to the treatment planning. Compared to the poly(ethylene-*co*-cyclohexane 1,4-dimethanol terephthalate) films widely used in orthodontic treatment all over the world [43], the TPU film material in this article exhibited higher rigidity (Fig. 3), i.e. more than twice the elastic modulus value of the reference material, and high yield stress and excellent elongation as well. The combination of balanced high rigidity and ductility give this material better teeth movement controlling ability and durability, which is crucial in managing the comprehensive orthodontic treatment, such as cases of tooth extraction, which is very common among Asians.

The TPU film used in this study had good stress-relaxation property (Fig. 3), which is another critical issue to the orthodontic treatment since the orthodontic force provided by the clear aligners fabricated via this TPU film would be more sustainable and the effective teeth movement could last for a longer time. Polycarbonate, as another traditional material for clear aligners, might have biological risks since it may release bisphenol A during orthodontic process. Overall, our TPU film is balanced in mechanical properties and biocompatibility, which allows it to serve as an ideal clear aligner material.

The finite element analysis of the thermoforming process of the TPU film demonstrated the thickness distribution of the formed clear aligner (Fig. 4). Following the *in vitro* examinations of biocompatibility (Fig. 5) and *in vivo* animal experiments (Fig. 6), we have achieved clinical applications for 120 cases with the classification listed in Table 1. One out of the 120 cases is demonstrated in Fig. 7, which is a representative case of crowding treated by our clear aligners.

While both biomaterials [44–53] and 3D printing [54–55] have been much investigated, few of them can be perfectly combined in a real clinical translation. The article reports a clinical case using a thermoplastic polymeric biomaterial as clear aligners templated by 3D-printed thermosetting polymeric molds. With a growing number of people who begin to pay attention to the health and beauty of their teeth, the orthodontic treatment would gain more popularity within days. The clear orthodontic industry combines multiple branches of science and engineering like material science, orthodontics and computer science; along with the improvement of these science and technology, clear aligners would keep evolving into better orthodontic appliances.

## Conclusions

Polymeric clear aligners were fabricated after assembling 3D printing technology, biomaterials science and computer simulation. Following examinations of the key parameters of photocurable 3D printing of a resin, we successfully printed a series of dental models for the thermoforming of clear aligners. Finite element analysis was also performed to simulate the thermoforming process of the TPU film and plan the series of clear aligners for the orthodontic treatment. Both animal experiments and clinical cases illustrated the feasibility of the ‘invisible’ orthodontics by polymeric ‘clear’ aligners molded on the 3D-printed personalized dental models. Nevertheless, an ideal film material for clear aligners should be resistant to stress relaxation, tearing, swelling in wet environments, dyeing, and even bacterium, and it might be hard for a single layer film material to achieve an ideal material. Hence in the future, a multilayer film material with various functions might be promising to meet the orthodontics requirements.

## Supplementary data

Supplementary data are available at *REGBIO* online.

## Funding

The authors are grateful for the financial supports from NSF of China (grants No. 52130302 and 21961160721), National Key R&D Program of China (grant No. 2016YFC1100300).

*Conflicts of interest statement.* Y.Z. and L.H. are employees of EA Medical Device Technologies Company. The other authors declare no conflict of interest.

## Supplementary Material

rbac007_Supplementary_Data
