# Supplementary material for: ‘Invisible’ orthodontics by polymeric ‘clear’ aligners molded on 3D-printed personalized dental models
Source: Regen Biomater. 2022 Feb 4;9:rbac007. doi: 10.1093/rb/rbac007 (PMC8992363; doi:10.1093/rb/rbac007)
Supplement: rbac007_Supplementary_Data [file rbac007_supplementary_data.docx]

SUPPORTING INFORMATION

**“Invisible” orthodontics by polymeric “clear” aligners molded on 3D-printed personalized dental models**

Xiaoye Yu^1^, Guanghui Li^2^, Yikan Zheng^3^, Jingming Gao^1^, Ye Fu^1^, Qunsong Wang^1^, Lei Huang^3,*^, Xiaogang Pan^2,*^, Jiandong Ding^1,*^

^1^ State Key Laboratory of Molecular Engineering of Polymers, Department of Macromolecular Science, Fudan University, Shanghai 200438, China

^2^ Department of Orthodontics, Shanghai Ninth People’s Hospital, College of Stomatology, Shanghai Jiao Tong University School of Medicine, National Clinical Research Center for Oral Diseases, Shanghai Key Laboratory of Stomatology & Shanghai Research Institute of Stomatology, Shanghai 200011, China

^3^ Angelalign Research Institute, Shanghai 200438, China

* Corresponding authors. Emails: jdding1@fudan.edu.cn (JD Ding), xgpan70@126.com (XG Pan), huanglei@angelalign.com (L Huang)

**Table S1.** Resin formula for 3D printing of the dental model

| Component | Code name | Fraction |
| --- | --- | --- |
| Epoxy acrylate oligomer | O-101 | 45% |
| Aliphatic urethane acrylate oligomer | O-102 | 10% |
| Multiple functional groups monomer | M-201 | 5% |
| Dual functional groups monomer | M-202 | 15% |
| Single functional group monomer | M-203 | 24% |
| Initiator | I-301 | 1% |


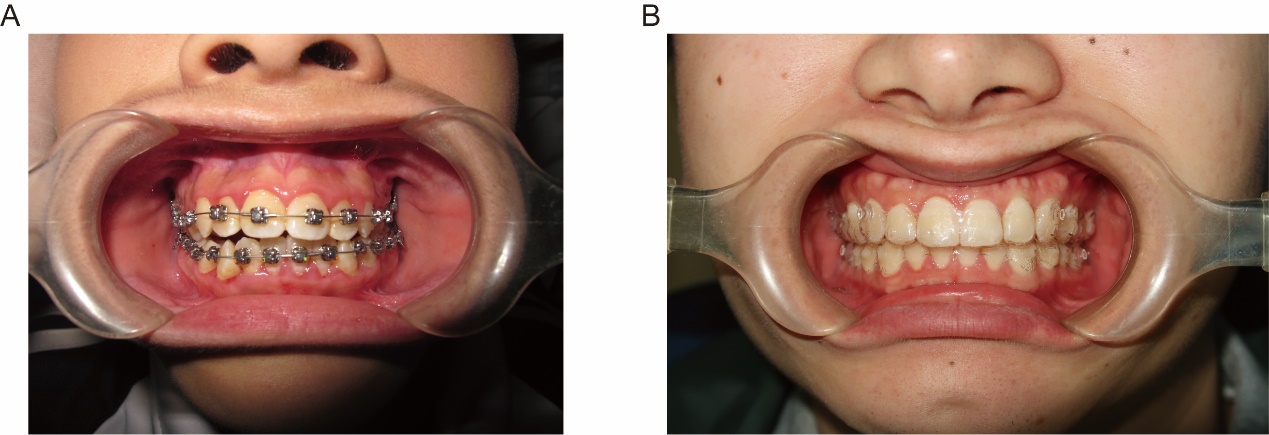


**Figure S1.** Photographs of patients’ oral cavities that treated with A) traditional metal braces and B) clear polymeric aligners.


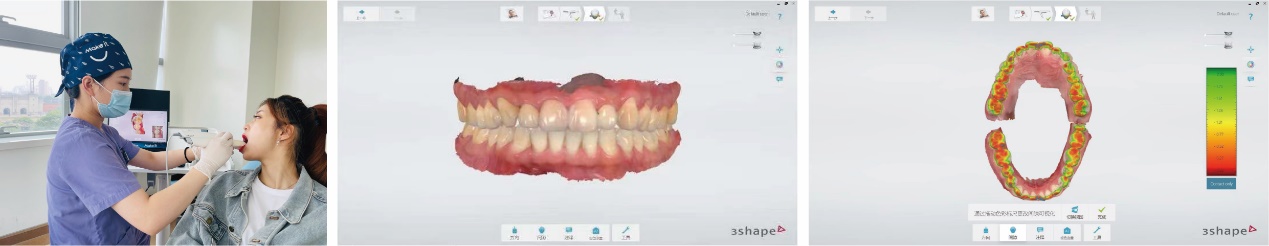


**Figure S2.** Demonstration of the oral cavity scanning and data processing.


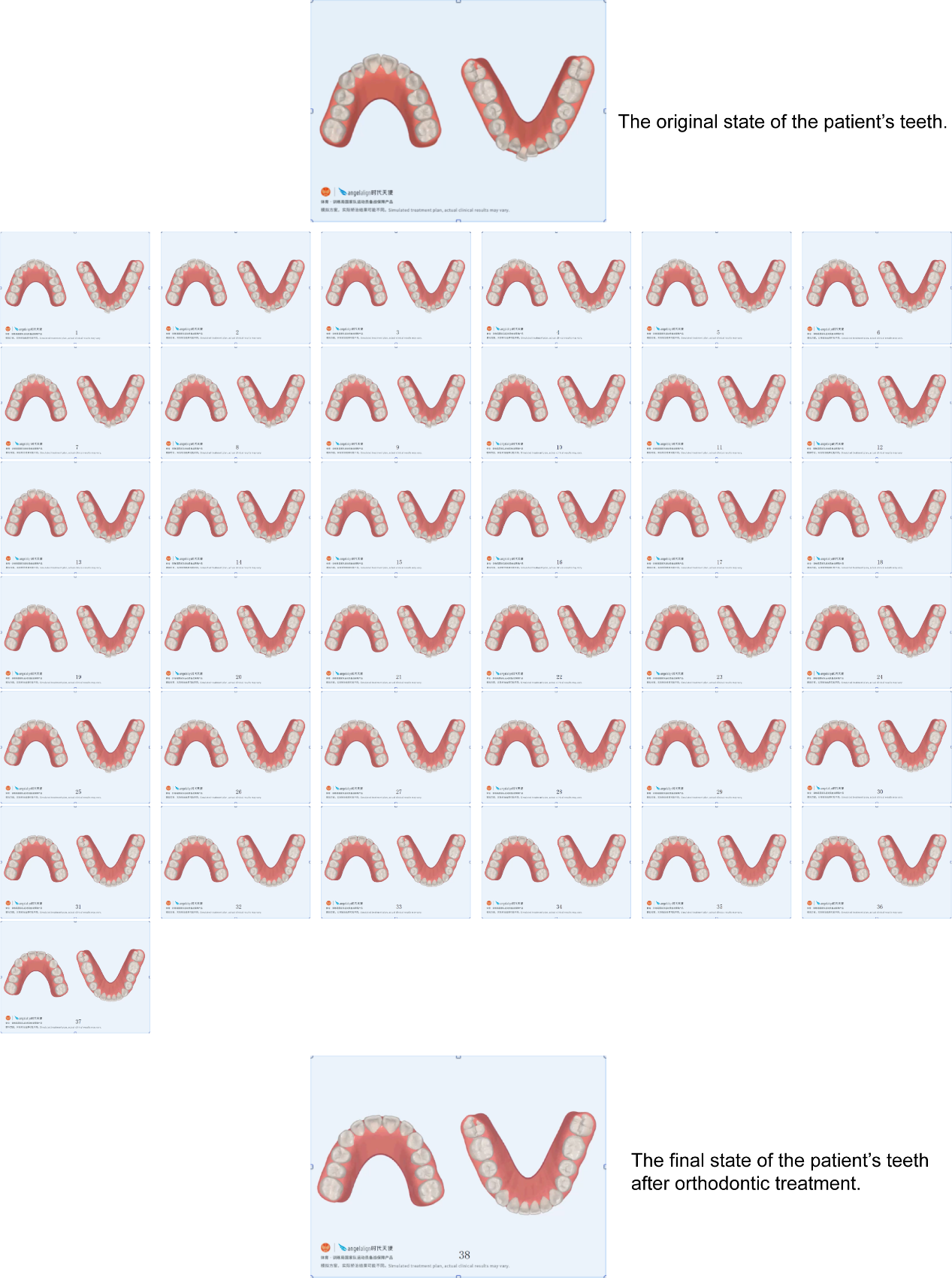


**Figure S3.** Computer virtual design outputs of all steps of the orthodontic treatment of a case for a 12-year-old girl.

**
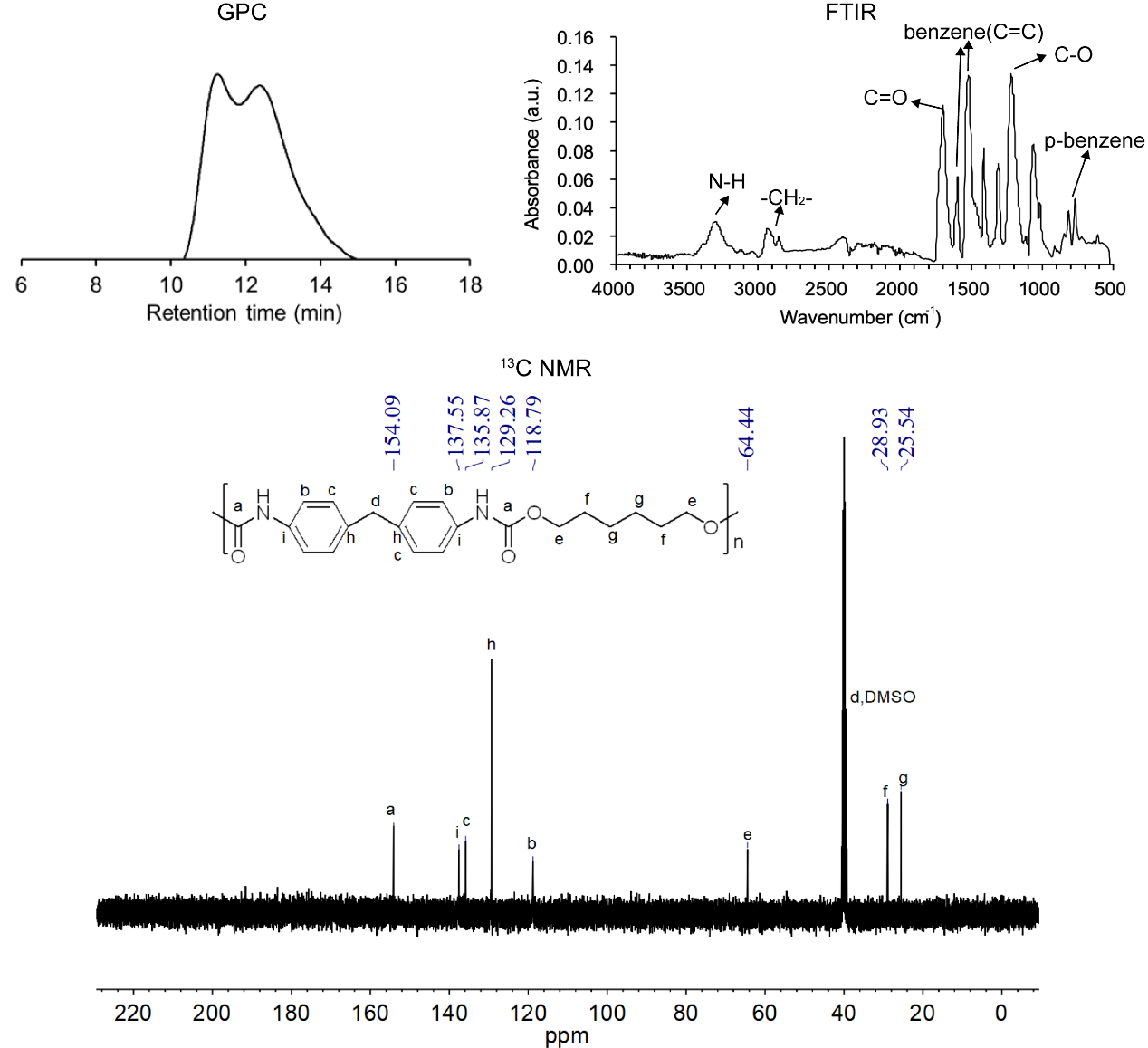
**

**Figure S4.** GPC curve, FTIR spectrum and ^13^C NMR spectrum of the thermoplastic polyurethane (TPU) film.

**
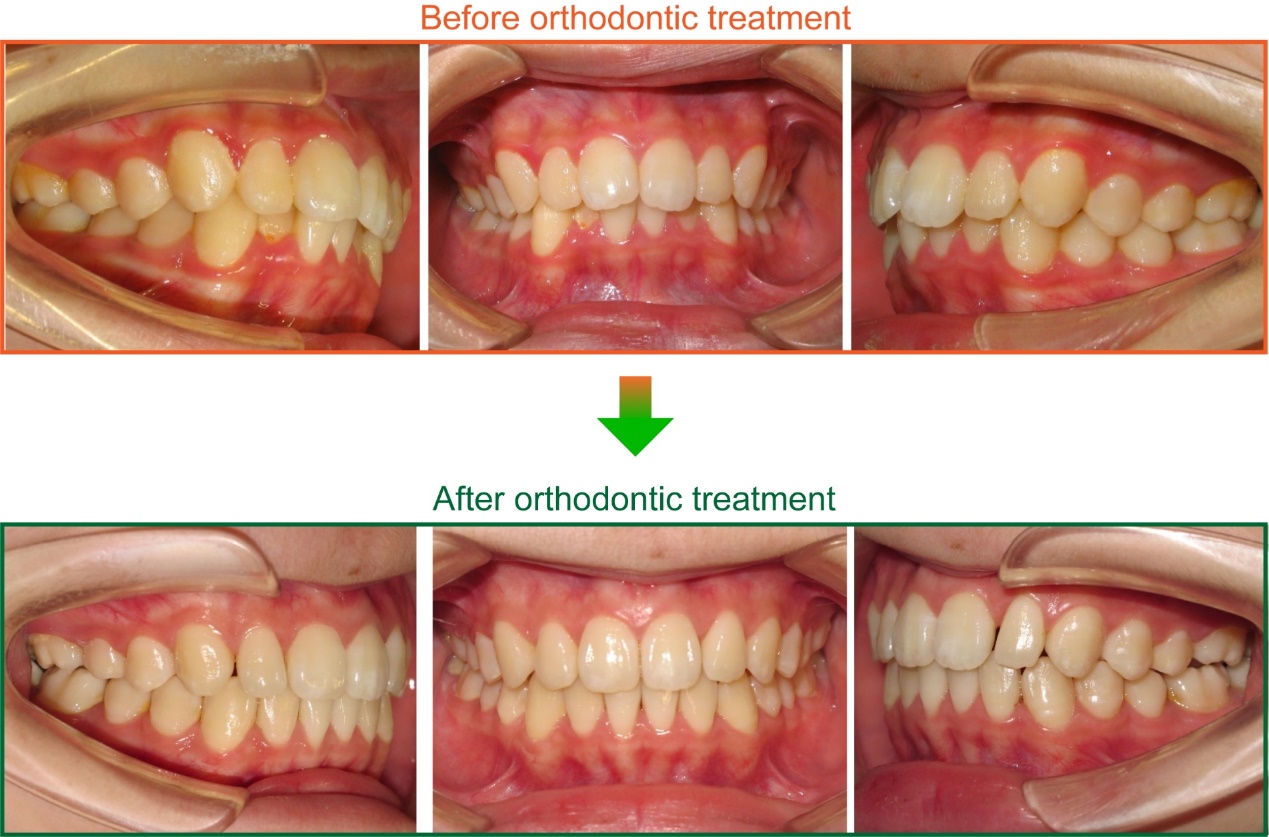
Figure S5.** Photographs of the dentition of the 12-year-old girl before and after the orthodontic treatment.


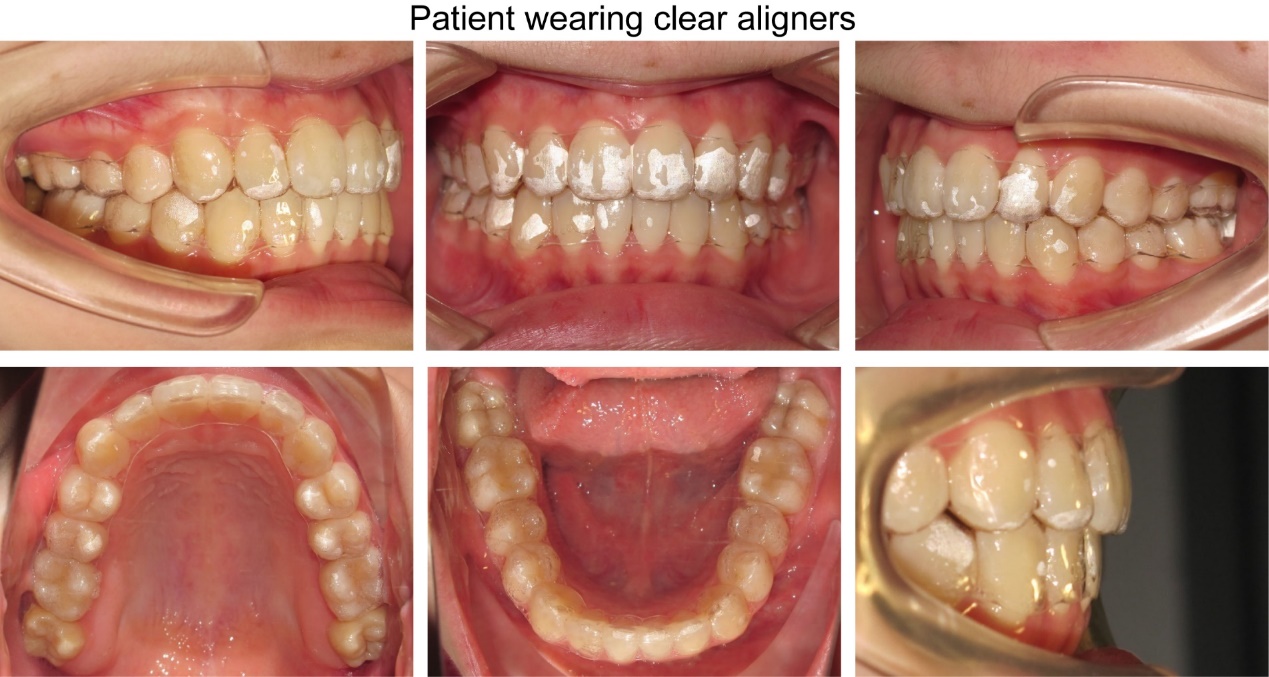
**Figure S6.** Photographs of the 12-year-old girl wearing the clear aligner.
